# Genomics of a pediatric ovarian fibrosarcoma. Association with the DICER1 syndrome

**DOI:** 10.1038/s41598-018-21663-9

**Published:** 2018-02-19

**Authors:** Jorge Melendez-Zajgla, Gabriela E. Mercado-Celis, Javier Gaytan-Cervantes, Amada Torres, Nayeli Belem Gabiño, Martha Zapata-Tarres, Luis Enrique Juarez-Villegas, Pablo Lezama, Vilma Maldonado, Karen Ruiz-Monroy, Elvia Mendoza-Caamal

**Affiliations:** 1 Functional Genomics Laboratory, Instituto Nacional de Medicina Genomica, Mexico City, Mexico; 2 Facultad de Odontologia, Universidad Nacional Autonoma de Mexico, Mexico City, Mexico; 3 Group of Reproductive Development and Apomixis, Laboratorio Nacional de Genómica para la Biodiversidad (LANGEBIO), CINVESTAV, Irapuato, Guanajuato, Mexico; 4 Histopathology Unit, Instituto Nacional de Medicina Genomica, Mexico City, Mexico; 5 Hospital Infantil de Mexico, Mexico City, Mexico; 6 Instituto Nacional de Pediatria, Mexico City, Mexico; 7 Epigenomics Laboratory, Instituto Nacional de Medicina Genomica, Mexico City, Mexico; 8 Clinical Area, Instituto Nacional de Medicina Genomica, Mexico City, Mexico

## Abstract

Ovarian fibrosarcomas are extremely rare tumors with little genomic information available to date. In the present report we present the tumoral exome and transcriptome and the germinal exome of an ovarian fibrosarcoma from a 9-years old child. We found a paucity of mutations (0.77/Mb) and CNV alterations. Of these, the most relevant were a point mutation in the metal-binding site of the microRNA-processing DICER1 enzyme and a frame-shift alteration in the tumor suppressor gene NF1. We validated a germinal truncating mutation in DICER1, which was consistent with a DICER1 Syndrome diagnosis, providing the first example of an ovarian fibrosarcoma as the presenting neoplasia in this syndrome. Network and enrichment analyses showed that both a mesenchymal signature and a Hedgehog cascade could be driving the progression of this tumor. We were also able to find a global lincRNA deregulation, as the number of lincRNAs transcripts expressed in the tumor was decreased, with a concomitant upregulation of previously described non-coding transcripts associated with cancer, such as MALAT1, MIR181A1HG, CASC1, XIST and FENDRR. DICER1 Syndrome should be considered as a possible diagnosis in children ovarian fibrosarcoma. The role of lncRNAs in neoplasias associated with DICER1 alterations need to be studied in more detail.

## Introduction

Ovarian fibrosarcomas are rare and aggressive tumors. These sex cord-stromal neoplasias are derived from the stromal component of the ovary and give rise to large, highly vascular and mitotically-active tumors. Ovarian fibrosarcomas usually present in older adults, with a median age of presentation of 49 years, sometimes associated to co-morbidities such as Maffucci´s syndrome^1^, naevoid basal cell carcinoma syndrome^2^, or other congenital syndromes^3^. Less than 5% of the reported cases are under 10 years old, including a case in an 8-year-old girl with nevoid basal-cell carcinoma syndrome^2,4,5^. Very little is known about the molecular characteristics of these tumors.

Recently, germline truncating mutations in the microRNA-processing protein DICER1 gene have been reported in patients with pleuropulmonary blastoma (PPB) or the related familiar DICER1 syndrome which includes, besides PPB, cystic nephroma, Sertoli-Leydig cell tumors, medulloepitheliomas and embryonal rhabdomyosarcomas^6^. In addition, somatic mutations in the RNAse IIIb domain have been also found in a discrete number of tumors, being common only in non-epithelial ovarian cancers, in which the prevalence can be as a high as 60%^7^. MicroRNAs are small non-coding RNAs that regulate the degradation of messenger RNAs. MicroRNA precursor (pre-miRNAs) transcripts are cleaved by the endoribonuclease Dicer1 into mature miRNA. Dicer´s RNAse IIIb domain cleaves the 5′-arm of the pre-miRNA and loads it to the RNA-induced silencing complex (RISC)^8^. It has been previously shown that DICER1 acts via haploinsufficiency since the lack of full DICER1 activity contributes to oncogenesis, without the need of a “hit” in the second allele^9^. Nevertheless, recent reports suggested that DICER1 could be acting as an oncogenic driver. In this model, an initial loss of the germinal DICER1 activity in patients with DICER syndrome would make primitive cells susceptible to oncogenesis by a second mutation in a RNAse IIIb “hotspot”, altering microRNA processing^10^.

Due to their rarity, there are no deep characterizations of the molecular alterations in these ovarian fibrosarcomas. In the present article, we present the first genomic report of this rare neoplasia from a 9-years old child and provide evidence that this neoplasia needs to be added as a presentation tumor in DICER1 syndrome patients, supporting an oncogenic role for this gene.

## Results

### Exome sequencing of paired samples shows a low rate of mutations

**Figure 1:**
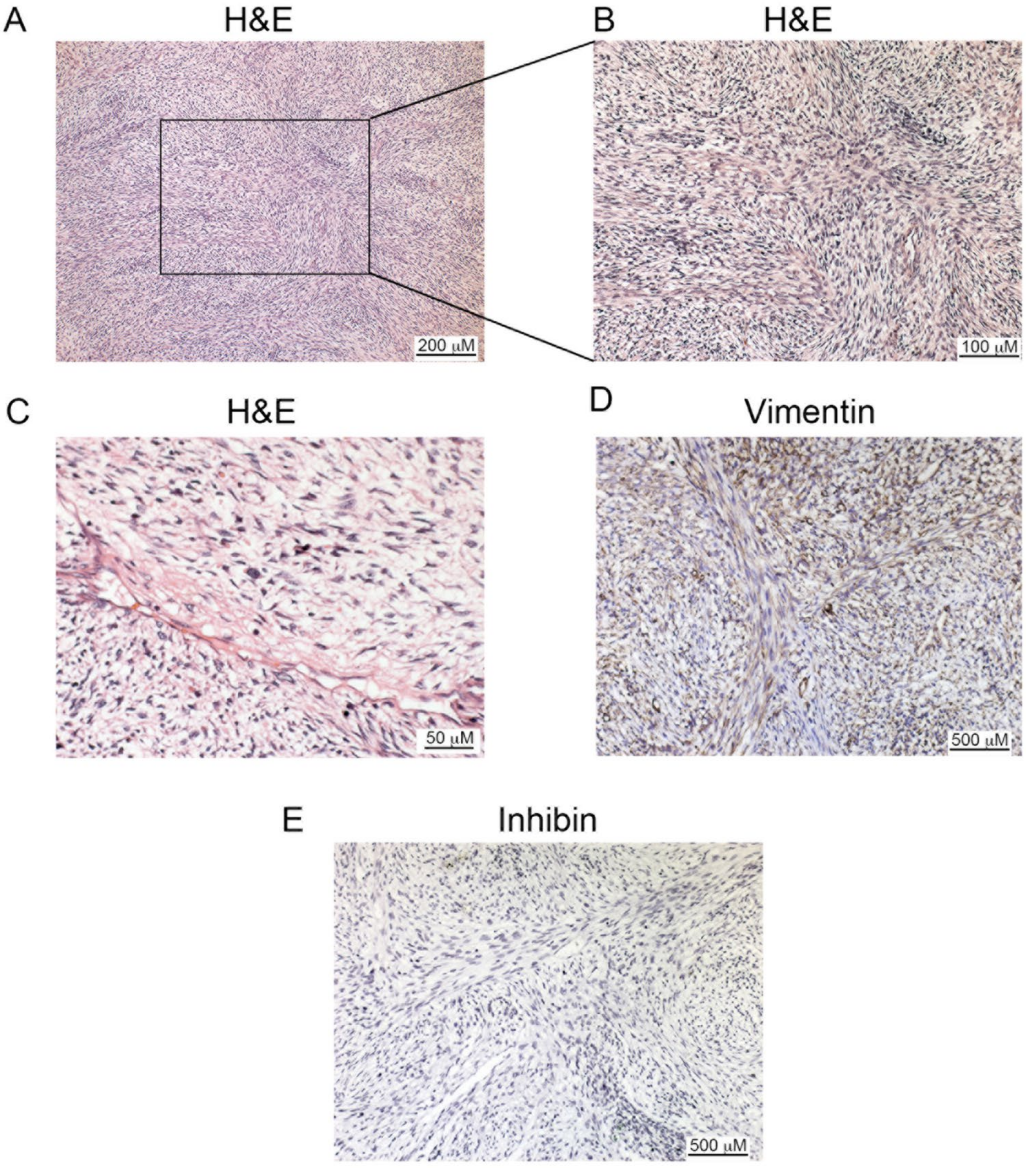
Ovarian fibrosarcoma pathology. (**A**) Hematoxylin-Eosin staining at 40x, (**B**) 100x and (**C**) 200x. (**D**) Immunohistochemistry of Vimentin. (**E**) Immunohistochemistry of Inhibin. Scale bars are shown at the right bottom of each micrograph.

**Figure 2:**
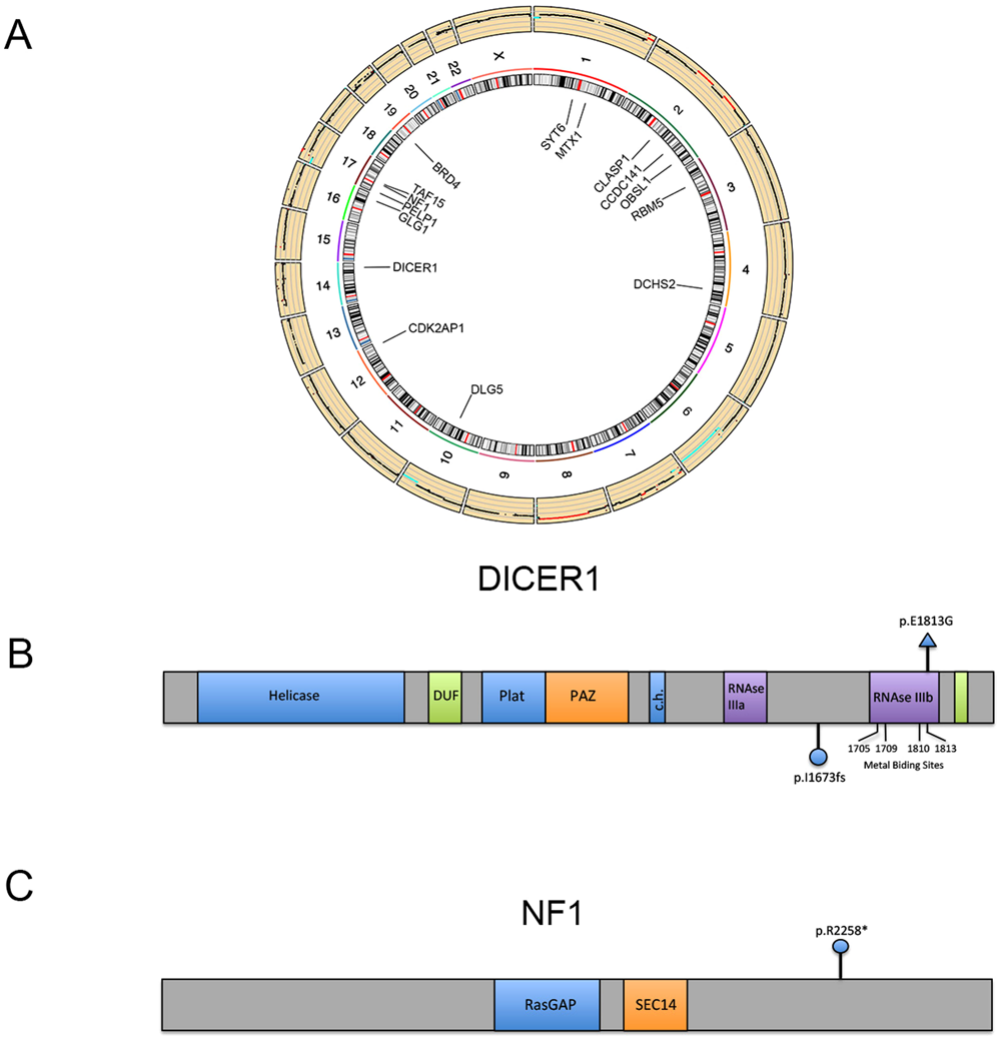
Mutational landscape of the ovarian fibrosarcoma. (**A**) Circos plot showing, at the outermost ring, the copy-number alterations found in the tumor. Mutations (SNV and InDels) in codifying genes are shown at the center. (**B**) Diagram of the DICER1 gene, showing its main domains. The mutation found in the tumor is marked at the upper side of the diagram, whereas the mutation found in the germinal DNA is depicted below it. (**C**) Diagram of the NF1 gene, showing its main domains. The mutation found in the tumor is shown.

A 9-year-old girl, without significant pathologic antecedents, received a diagnosis of an ovarian fibrosarcoma supported by morphology (Fig. 1A–C), and a positive immunohistochemistry staining for vimentin (100% positive cells) and negative for inhibin (Fig. 1D,E). In order to get insight into the genomic alterations of the rare pediatric tumor, we sequenced paired exomes of tumor and normal (white blood cells) tissues from the patient. We obtained 34 single nucleotide variants (SNV) and 286 small insertions and deletions (InDels) (Supplementary Data). The low mutation rate (0.77/Mb) found is typical of pediatric tumors, as reported elsewhere^11^. We then created a high confidence (HC) gene list that included only mutations in coding regions with a high probability of being deleterious, as assessed by the CADD algorithm^12^ (see Methods). Figure 2A and Table 1 shows that 16 mutations complied with the aforementioned criteria. To validate these mutations, we employed RNASeq from the tumor samples. We were able to corroborate 5 out of the 16 HC mutations. Most of the remaining mutations presented a very low or absent expression in the tumor or, alternatively, low allelic fraction in the initial exome analysis, possible due to clonal heterogeneity. Nevertheless, we were able to validate expressed mutations in two previously reported cancer drivers: DICER1 and NF1. DICER1 gene presented a missense mutation predicted to change a glutamic acid for a glycine in the residue 1813 of the protein (Fig. 2B). Interestingly, mutations in this metal-binding site abolish the RNAse IIIb activity of the enzyme, changing the specificity of its microRNA-processing ability^13^. Mutations in this site have been found in other tumor types^7^, although no alterations have been described in fibrosarcomas. We also validated a frame-shift mutation in NF1, a tumor-suppressor gene that is involved in the generation of multiple subtypes of soft-tissue sarcomas^14^ and has been associated with poor response to chemotherapy and targeted therapy^15^. This mutation is predicted to produce a functional truncated protein (Fig. 2C). We could not find a second alteration in the remaining allele of this gene. Nevertheless, the expression levels of its main isoform, ENST00000356175.7_NF1-002 were significatively downregulated (Fold change: 0.045, PPEE = 0).

**Table 1:** Filtered SNV and InDel from the tumor exome.

Gene	Variation class	Variation type	Effect	AF
BRD4	Missense Mutation	SNV	p.A1243D	0.333
CCDC141	Missense Mutation	SNV	p.K992T	0.123
CDK2AP1	Missense Mutation	SNV	p.R99K	0.098
CLASP1	Missense Mutation	SNV	p.R276L	0.426
DCHS2	Missense Mutation	SNV	p.R799H	0.185
DICER1	Missense Mutation	SNV	p.E1813G	0.435
DLG5	Nonsense Mutation	SNV	p.R530*	0.394
GLG1	Splice Site	SNV	p.P147L	0.106
MTX1	Frame Shift Del	DEL	p.F157fs	0.714
NF1	Nonsense Mutation	SNV	p.R2258*	0.069
OBSL1	Missense Mutation	SNV	p.R994C	0.161
PELP1	Frame Shift Del	DEL	p.P1025fs	0.333
RBM5	Missense Mutation	SNV	p.G729S	0.457
SCARF1	Frame Shift Ins	INS	p.G303fs	0.250
SYT6	Missense Mutation	SNV	p.S404V	0.244
TAF15	Missense Mutation	SNV	p.R165L	0.420

Del: Deletion. SNV: Single Nucleotide Variation. AF: Allelic Frequency.

### Copy number variation analysis

**Figure 3:**
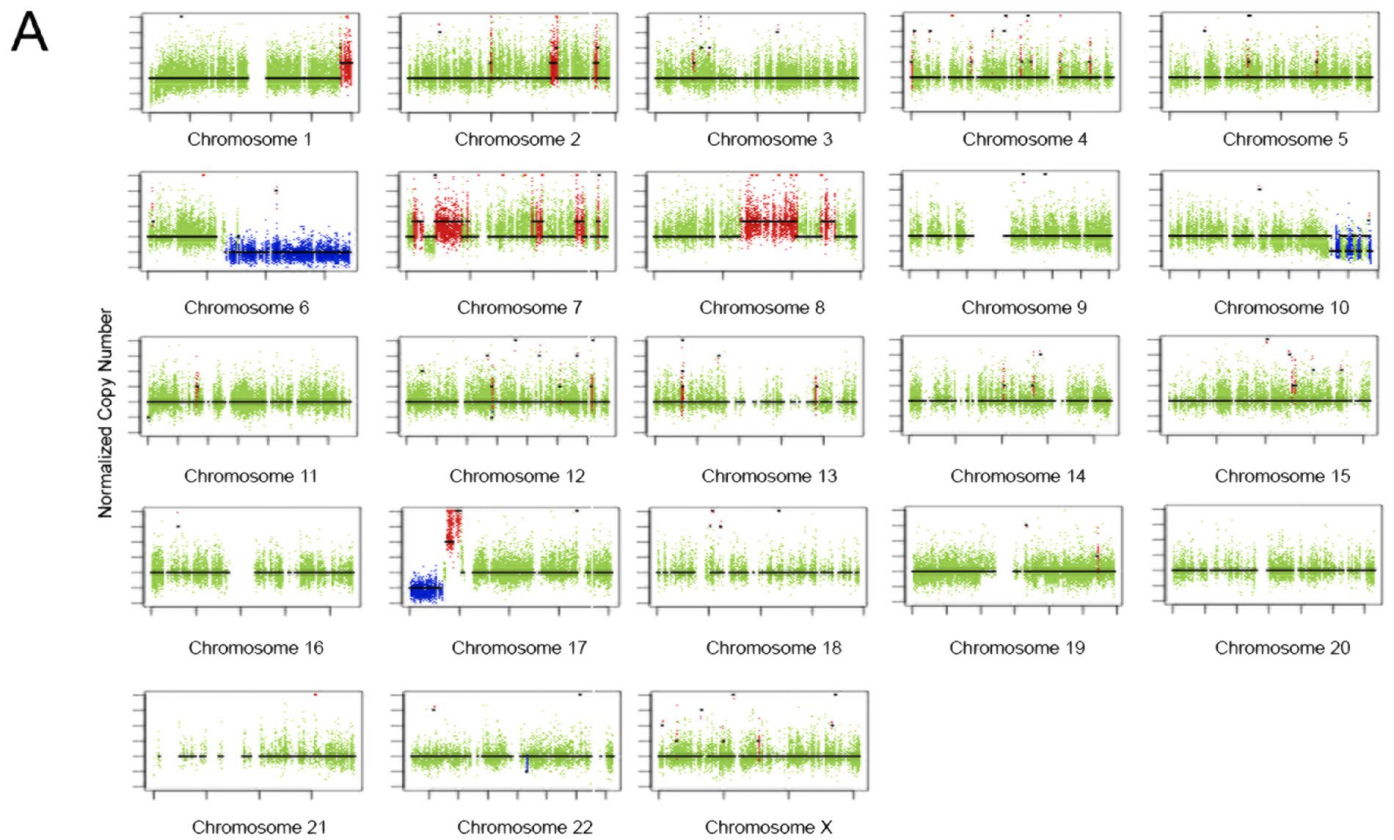
Copy number alterations in the ovarian fibrosarcoma sample. (**A**) Copy-number profile in the tumor. The normalized copy number is depicted against the length of each chromosome. The mean copy number is marked with a line. Deletions are depicted below and amplifications above the mean copy number line.

We then analyzed the data for the presence of Copy Number Variants (CNV). Figure 3A shows the presence of amplifications in large regions of chromosomes 1, 2, 7, 17 and, more important, large regions of chromosome 8. It has been shown that chromosome 8 trisomy is a marker that distinguishes ovary fibromas from fibrosarcomas^16^. We also found large deletions affecting the long arm of chromosomes 6 and 10 and the short arm of chromosome 17. To obtain a pathologically relevant CNV gene list we filtered the genes within the variant regions against a curated cancer drivers list^17^. Table 2 shows the genes affected by CNV in the tumor. Interestingly, we found amplifications of MYC, which have been commonly reported for sarcomas^18^ and deletions of TP53, another common finding in these tumors^19^. Interestingly, we found an increase in the expression of c-Myc when compared to normal ovary samples (283 vs 160 FPKM), although it did not reach significance.

**Table 2:** Copy number variation in the tumor exome.

Gene	Effect	Genotype
ARID1B	Deletion	0:−1
BRAF	Amplification	3:AAB
CASP8	Amplification	4:AAAB
EZH2	Amplification	3:AAB
FGFR2	Deletion	0:−1
IDH1	Amplification	3:AAB
MAP2K4	Deletion	0:−1
MET	Amplification	3:AAB
MLL3	Amplification	3:AAB
MYC	Amplification	3:AAB
NCOR1	Amplification	8:AAAAAABB
NFE2L2	Amplification	3:AAB
PRDM1	Deletion	0:−1
SF3B1	Amplification	4:AAAB
SMO	Amplification	3:AAB
TNFAIP3	Deletion	0:−1
TP53	Deletion	0:−1

Genotype: Allele number/Allele genotype. NC: Not calculated.

#### Mutations in non-coding RNAs

We also found several mutations in non-coding regions, including annotated lincRNAs (Table S2). None of these mutations have been reported in recent series of recurrent lincRNAs alterations in cancer^20^.

#### Micro-satellite instability status

In a recent survey of cancer cell lines, it was reported that 4 of 781 of these lines presented a DICER1 truncating mutation. Interestingly, all of them presented also microsatellite instability (MSI)^9^. For this reason, we analyzed the MSI status in this patient using the MSIseq package^21^ and found that the tumor indeed presented an MSI-High profile, being negative to a POLE-mutated phenotype. To validate this finding, we performed PCR and capillary electrophoresis of five commonly used MSI markers (BAT25, BAT26, NR21, NR22 and NR24) and found micro-satellite instability in three of the five markers analyzed (Table 3). We could not find mutations in MLH1, MSH2, MSH6 or PMS2 genes, although we cannot exclude a possible epigenetic mechanism of inactivation of MLH1 as the reason for the MSI phenotype.

**Table 3:** MSI markers.

Marker	Germinal size	Tumor size
BAT25	145 bp	140 bp
BAT26	140 bp	132 bp
NR21	120 bp	120 bp
NR22	160 bp	164 bp
NR24	150 bp	146 bp

The size of each marker is depicted for the germinal and tumor sample in base pairs (bp).

### Ovary fibrosarcoma’s transcriptome presents a mesenchymal network signature and an enriched Sonic Hedgehog network

**Figure 4:**
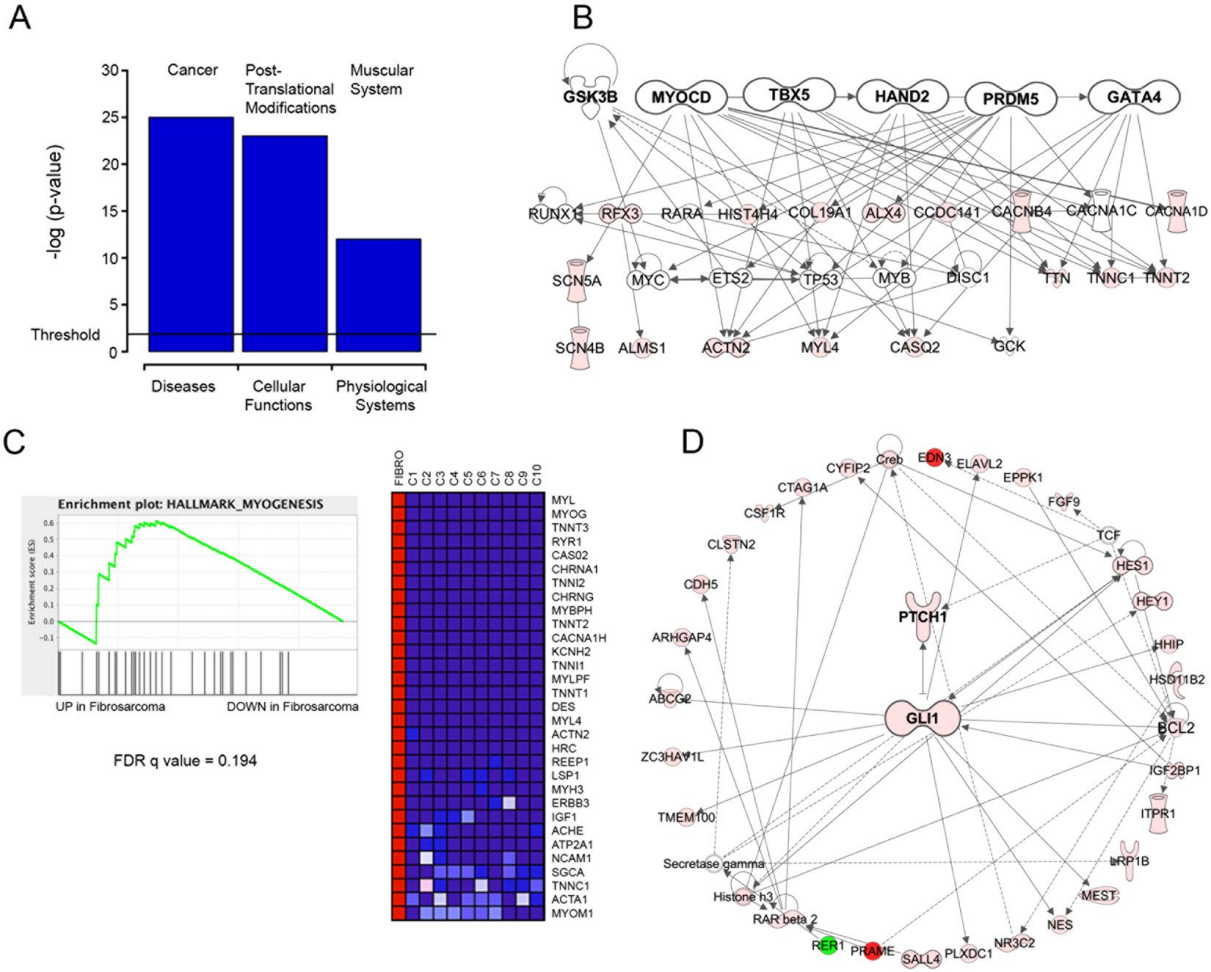
Network analysis of expression data. (**A**) The top enriched function in each category is plotted with the log p-value. The significance threshold is marked. (**B**) Mesenchymal network derived from the upstream/master regulators found in the expression dataset. (**C**) Gene Set Enrichment Analysis (GSEA) of the mesenchymal hallmark signature. Left Panel: The enrichment score is compared with the up-regulated (left) and down-regulated (right) genes. The False Discovery Rate is also shown. Right Panel: The top regulated genes found in the analysis. Lighter gray, higher expression. Darker gray, lower expression. (**D**) The Hedgehog pathway is enriched in the ovarian fibrosarcoma sample. Gray tones represent expressional changes.

We then compared the transcriptome data with a panel of previously reported normal fibroblast RNASeq experiments. We found 1193 Differentially-Expressed (DE) genes (Posterior Probability of Differential Expression (PPDE) >0.95 and Fold Change > 2) (Supplementary Data). To get more insight into the relevance of these transcriptional events, we performed a model network analysis using the Ingenuity Pathway Analysis tool (IPA, Qiagen Redwood City). Fig. S1 shows the enrichment of several processes on three main categories (Diseases, Molecular and Cellular Functions and Physiological Systems Functions). As expected, we found that the top enrichment in the Diseases category was Cancer, whereas postranslational modifications and muscular system were the most significant finding in molecular and physiological functions, respectively (Fig. 4A). Interestingly, the muscular system enrichment was driven by a group of genes responsible for regulating cascades related to mesenchymal development, as supported by a network upstream regulator analysis showing that a group of mesenchymal master regulators (including MYOCD, TBX5 and HAND2, Table 4) were leading this transcriptional cassette (Fig. 4B).

**Table 4:** Upstream regulators.

Gene	p-value	Variation type	Effect
MYOCD	1.23E^−06^	SNV	p.A1243D
HAND2	3.93E^−05^	SNV	p.K992T
TBX5	3.93E^−05^	SNV	p.R99K
GATA4	1.74E^−04^	SNV	p.R276L
UPF2	3.64E^−04^	SNV	p.R799H
VEGFA	4.60E^−04^	SNV	p.E1813G
NEUROG1	5.44E^−04^	SNV	p.R530*
TENM1	5.57E^−04^	SNV	p.P147L
DRAP1	6.16E^−04^	DEL	p.F157fs
NMNAT1	8.93E^−04^	SNV	p.R2258*
MED12	1.04E^−03^	SNV	p.R994C
MYOC	1.17E^−03^	DEL	p.P1025fs
estrogen receptor	1.22E^−03^	SNV	p.G729S
NOTCH3	1.31E^−03^	INS	p.G303fs
GLI1	1.61E^−03^	SNV	p.S404V
ZNF217	1.86E^−03^	SNV	p.R165L

Del: Deletion. SNV: Single Nucleotide Variation.

An additional analysis using the Gene Set Enrichment Analysis (GSEA) algorithm^22^ also revealed the enrichment of a myogenesis signature (Fig. 4C), further supporting the involvement of a mesenchymal transcription signature in the ovarian fibrosarcoma progression. Among the top regulated networks, we also found a GLI1-driven cascade (Fig. 4D), which could be one of the key pathways involved in this neoplasia, due to the previous report of mutations in members of the Sonic Hedgehog pathway and the involvement of GLI1 as one of the top upstream regulators discovered in our gene set (Table 4). We also analyzed the data for the presence of fusion transcripts, but we were unable to validate the positive hits found.

#### microRNAs deregulation in ovarian fibrosarcoma

We next sought to assess the microRNAs expression landscape in the fibrosarcoma sample. As expected, we found that a larger number of pre-miRNAs were deregulated, when compared to mature miRNAs (Fig. S1A). The top networks associated with the regulated genes were associated with MYC and the Argonaute proteins AGO1 and AGO3 (Fig. S2B and Supplementary Data).

#### lncRNAs deregulation in ovarian fibrosarcoma

**Figure 5:**
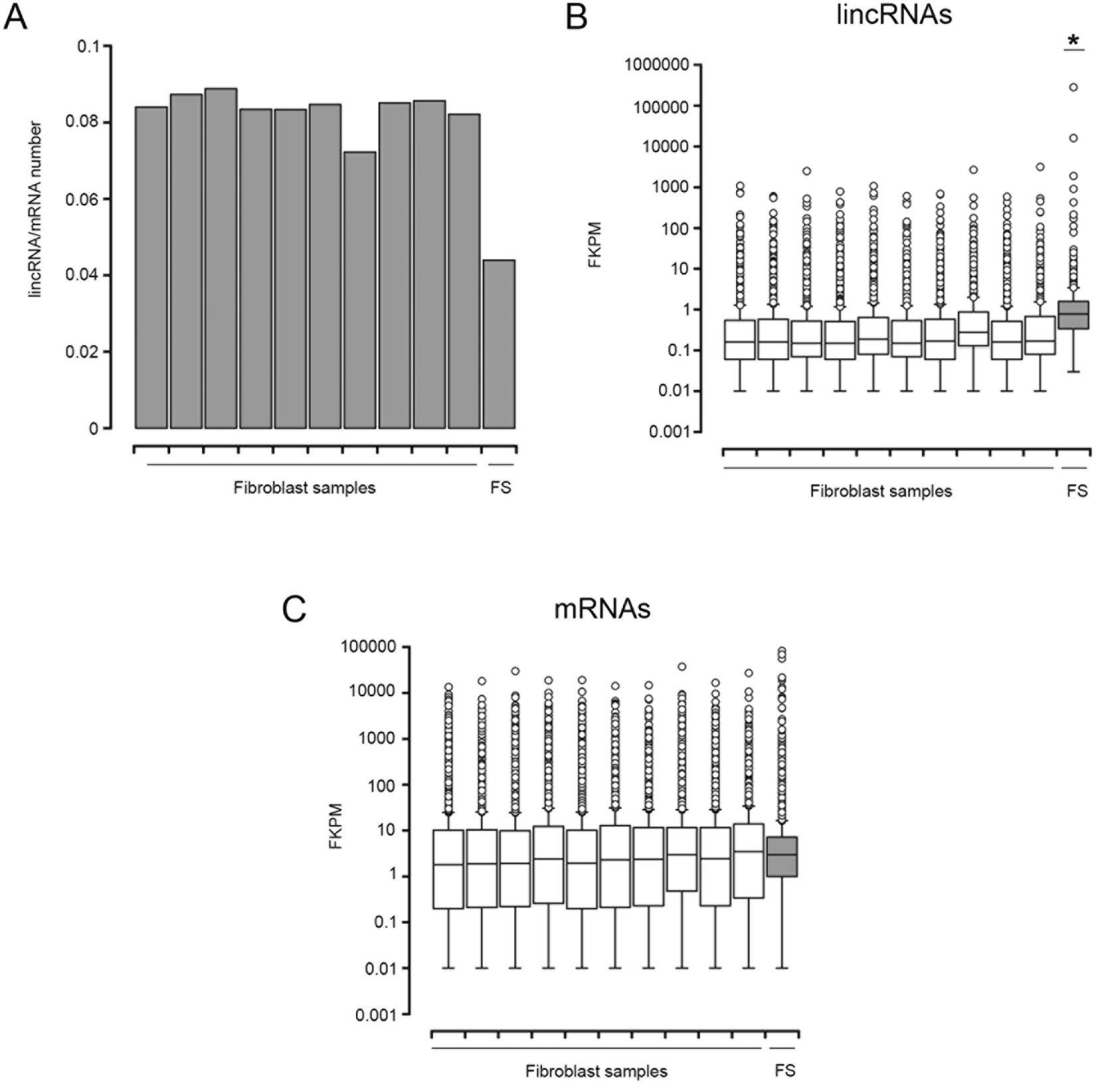
Long-intergenic non-coding RNAs are deregulated in the fibrosarcoma sample. (**A**) Plot showing the ratio of lincRNAs versus mRNAs in the fibrosarcoma (FS) and control fibroblasts samples. (**B**) Boxplots showing the lincRNAs expression, in Fragments Per Kilobase of Exon per Million Fragments Mapped (FPKM), of each analyzed sample. The median, upper and lower quartiles and outliers are depicted for the fibrosarcoma (FS) and control fibroblasts samples. (**C**) Boxplots showing the mRNAs expression in FPKM, of each analyzed sample. The median, upper and lower quartiles and outliers are depicted for the fibrosarcoma (FS) and control fibroblasts samples.

It has been recently described that DICER1 is able to control hundreds of long non-coding RNAs in a genome-wide fashion^23^. In this study, gene deletion or mutation of the RNAse III catalytic residues of DICER1 impaired the expression of hundreds of lincRNAs in mouse embryonic stem cells by a c-Myc-dependent mechanism. To explore if this regulation could also be present in cancer, and in particular in the fibrosarcoma sample under study, we compared the ratio of expressed lincRNAs versus mRNAs in our sample with a panel of normal fibroblasts. Consistent with this hypothesis, we found a clear depletion in the number of expressed lincRNAs (Fisher exact test p < 2.2^−16^) and Fig. 5A. A similar depletion was found when we performed a comparison with panels of normal and tumoral ovary samples (Fisher exact test p < 2.2^−16^) Fig. S3A. Unexpectedly, lincRNAs expression was higher in the tumoral sample than in the control fibroblasts or normal ovary cell lines, pointing toward a specific regulation in this tumor (Figs 5B,C and S3). This is supported by the presence of several oncogenic lincRNAs in the top expressed and DE non-coding RNA genes (Table 5), including MALAT1, MIR181A1HG, CASC1, XIST and FENDRR. Interestingly, several tumor samples presented also higher lincRNAs expression and, coincidentally, these tumors tended to have higher (although statistically non-significative) DICER1 expression (Fig. S3).

**Table 5:** Differentially expressed lincRNAs.

lincRNA	PPDE	Fold Change
LINC01087	1.00	33072
LINC01266	1.00	46690
**XIST** ^39^	1.00	4464627
**MIR181A1HG** ^40^	1.00	817
**MALAT1** ^41^	1.00	35
RP11-384P7.7	1.00	377
RP11-701H24.4	1.00	93
**RMRP** ^42^	1.00	904540
**TSIX** ^43^	1.00	6021
**CASC15** ^44^	1.00	54
UG0898H09	1.00	3517
SCARNA2	1.00	3335
CH507-513H4.5	1.00	7249
RP11-693J15.5	1.00	665
LINC01597	1.00	262
RP11-923I11.6	1.00	55
CTD-2545M3.8	1.00	580
**FENDRR** ^45^	1.00	88
RP11-13K12.1	1.00	15564
**LINC00626** ^46^	1.00	2189
DKFZP434L187	1.00	863
LINC00184	1.00	55
RP11-268G12.1	1.00	925
PWAR6	1.00	17
**RP11-191L9.4** ^47^	1.00	11673
LINC00645	1.00	11673
AC083843.1	1.00	17
RP11-706O15.5	1.00	46
**FIRRE** ^48^	1.00	232
RP11-138A9.1	1.00	1625
LINC01579	1.00	122
**MIR99AHG** ^49^	1.00	34
RP11-244M2.1	1.00	38
RP1-78B3.1	1.00	104
AC096669.3	1.00	9728
RP11-13K12.5	1.00	9728
CTA-280A3.2	1.00	9728
RP11-348P10.2	1.00	13
**NBAT1** ^50^	0.99	72
**RP11-161M6.2** ^51^	0.99	46
RP11-448A19.1	0.99	10
PKIA-AS1	0.99	258
CTC-444N24.8	0.97	6
CTD-2311B13.1	0.97	7782
RP11-453M23.1	0.97	7782
FAM95C	0.97	7782
RP11-37B2.1	0.96	5

PPDE: Posterior probability of differential expression. LincRNAs involved in cancer are marked in bold, with the associated reference.

### Germinal mutations analysis confirms DICER1 Syndrome

**Figure 6:**
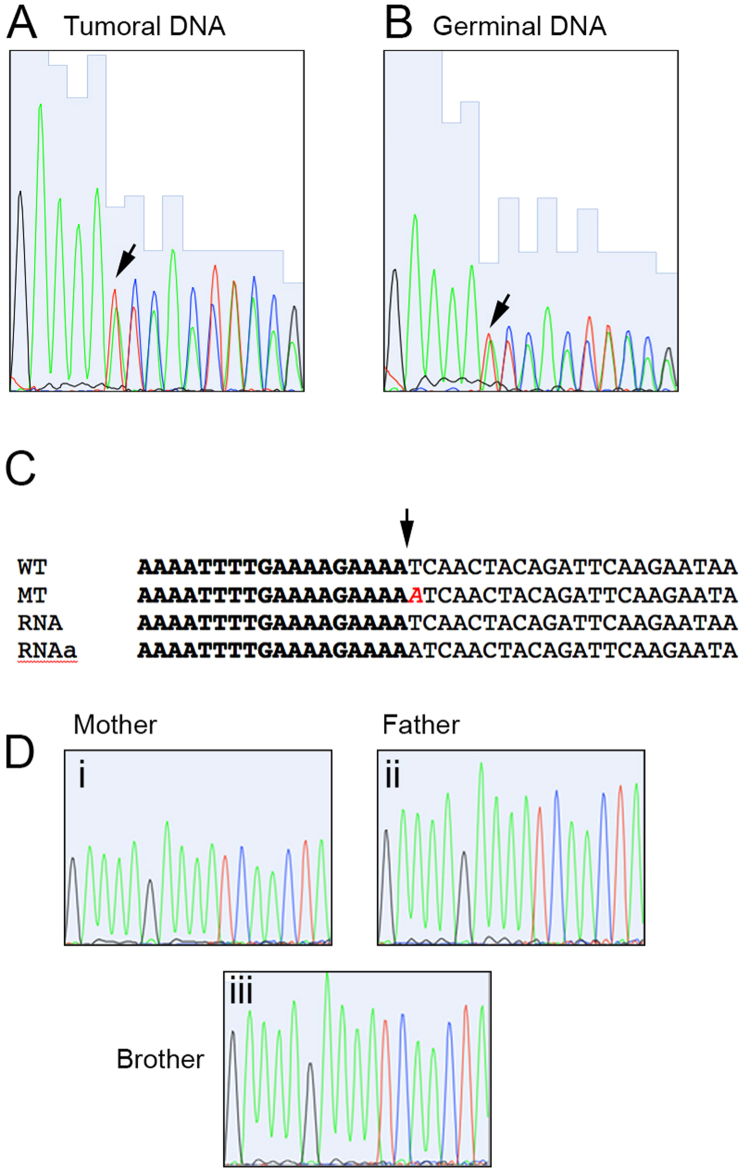
DICER1 mutations. Sequence traces from Sanger sequencing of the (**A**) Tumoral and (**B**) Germinal DNA. The arrow shows the insertion point, where a duplicate signal starts. (**C**) Nucleotide alignment of wild type (WT) and mutated (MT) sequences derived from the exome data, with the sequences obtained from RNA (RNA and RNAa). The inserted adenine is shown in cursive. (**D**) Sequence traces from the mother (i), father (ii) and brother (iii) of the affected individual.

We then analyzed the germinal line for putative pathological variants that could be accounting for the early presentation of this rare tumor. We established a tiered classification, after filtering variants using the criteria stated in Methods. We were able to find two tier 1 mutations (mutations in genes previously reported as dominant-acting in pediatric hereditary tumors) in our data. Of these mutations, the PALB2 alteration has been reported as likely benign in ClinVar (ID 142310) so we excluded it as a pathogenic contributor. Remarkably, the remaining mutation affected DICER1 gene. This mutation, not been reported in public databases, consisted in a frame shift insertion, predicted to produce a functional truncated protein (Fig. 2B). This finding was verified by Sanger sequencing in both tumoral and germinal DNA, as well as in RNA derived from the tumor (Fig. 6A–C and not shown). Although the MuTect algorithm called a dinucleotide insertion, these assays showed a single-base insertion. The presence of a DICER1 functional truncating germinal mutation associated with an additional tumor mutation event in the RNAse IIIb domain of this enzyme is characteristic of the associated neoplasias characteristic of the recently described DICER1 Syndrome (OMIM 601200)^9^. To validate this finding, we sequenced the altered region in germinal DNA derived from the parents and a sibling. Figure 6D shows that only the proband presented the mutation in DICER, pointing toward a *de novo* mutation in this gene as the cause for the syndrome, although we cannot exclude germline mosaicism in one of the parents.

## Discussion

In the present article, we report the genomic characterization of a fibrosarcoma arising from the ovary of a 9 years-old child. It has been previously reported that adult ovary fibrosarcomas present structural alterations, including chromosomal number aberrations such as trisomy of the 12 and 8 chromosomes. Trisomy 8 has been postulated as a marker for distinguishing fibroma from fibrosarcoma^16^. In the present report we were able to show higher copy number of large regions in chromosome 8, consistent with its proposed role as a fibrosarcoma marker.

MYC is one of the most recurrent alterations in sarcomas^18^. A recent report has shown that a DICER1-microRNA-Myc circuit is responsible for the steady-state transcription of hundreds of long non-coding RNAs^23^. Since it has been reported lncRNAs are important in the progression of pediatric sarcomas^24^, it is tempting to speculate that the amplification of MYC could be required for fibrosarcoma progression in the specific setting of DICER1 germinal inactivation. In this report, the authors found that DICER1 RNAse III domain is responsible for the expression of a large number of these non-coding RNAs, in particular those responsible for maintaining pluripotency. This model closely resembles the progression of DICER1 Syndrome tumors, where an initial truncating mutation is later complemented with a second point mutation in the RNAse IIIb domain, giving rise to a diverse array of tumors, including the new association we report here. In their experiments, the authors found that the effect of DICER1 knockout/mutation depends on the regulation of a specific miRNA (miR-295) and the upregulation of c-Myc. Interestingly, we found that c-Myc was amplificated in the fibrosarcoma sample, when compared to normal fibroblasts, although we could not detect a significative increase in its expression. The initial oncogenic insult could be the loss of one DICER1 allele, which could change the expression of a subset of oncogenic lincRNAs that may drive the oncogenic initiation. The second and more specific loss would require the amplification of c-Myc to further drive cancer progression, in order to have minimal Myc expression levels. *In vitro* and *in vivo* models testing this are clearly needed to test this hypothesis.

In our data, we found a paucity of mutations, as reported for other pediatric tumors. The main possible drivers for this neoplasia were DICER1, NF1 and MYC. The first two presented mutations, whereas the latter two were altered by structural changes. As mentioned, we did not found alterations in the previously reported PTCH1 or Sonic Hedgehog pathway, although we cannot discard that epigenetic changes could be participating in the progression of the disease. This is supported by the transcriptome results, in which a significative deregulation of this pathway was found. DICER1 mutations are rare in cancer patients. Most of them are associated with infrequent embryonal or primitive tumors such as sex cord-stromal tumors of the ovary, embryonal rhabdomyosarcomas, Wilms tumors, etc., pointing toward a specific development pathway requirement for its cancer driver´s activity. More important, several of these mutations (although not all), are associated with germinal DICER1 mutations, in which the germinal allele generally presents a truncation mutation. Since no LOH has been found, and the second allele retains a modified activity, it has been proposed that DICER1 acts as an oncogene driver, with an altered microRNA processing activity acting as the oncogenic trigger^7^. A recent survey of cancer cell lines showed that 4 of 781 of these lines presented a DICER1 truncating mutation. All of them presented also microsatellite instability (MSI)^9^, so the authors conclude that DICER1 mutations were unlikely to be drivers. Nevertheless, in our patient the somatic mutation was found in a hot spot, consistent with its proposed oncogenic role. We tried to assert the reason for the MSI-H phenotype, but were unable to find mutations in MLH1, MSH2, MSH6 or PMS2 genes. Nevertheless, we cannot exclude a possible epigenetic mechanism of inactivation of MLH1.

## Methods

The project received ethical and scientific approval from Instituto Nacional de Medicina Genomica, Hospital Infantil de Mexico and Comision Federal para la Proteccion contra Riesgos Sanitarios (COFEPRIS) committees. After obtaining informed consent from the parents and the corresponding children assent, a sample derived from the surgical specimen was snap-frozen and a portion of it subjected to histopathological assessment. A blood sample was also obtained at the same time. The tumoral sample presented more than 80% neoplasic cellularity.

### Clinical Case

A 9-year-old girl, without a significant pathologic clinical history, was admitted at the Hospital Infantil de México Federico Gómez with one month´s history of moderate progressive abdominal pain localized in mesogastrium, vomiting and weight loss. A heterogeneous ovarian mass of 17 × 9,7 cm was discovered by CT scan image. The patient underwent surgical excision of the mass. On laparotomy, surgeons found a right ovarian tumor. The pathology specific presented a typical herringbone pattern, high mitogenic index (4 mitoses in 10 high-magnification fields), extensive hemorrhagic and necrotic areas and nuclear pleomorfism. The final histopathology diagnosis was fusocellular sarcoma compatible with fibrosarcoma. Immunohistochemistry was performed against Vimentin (MA5-11883, Invitrogen, CA, USA; 1:100 dilution) and Inhibin (5692, Bio SB, CA, USA; 1:100 dilution) as reported previously^25^. The patient had two siblings, her brother was diagnosed with bilateral renal tumors: Wilms´ tumor and a possible metanephric adenoma whereas her sister is healthy at the moment. We were unable to obtain paternity tests.

### Exome analysis

Genomic DNA from the patient was extracted using commercially available kits from primary tumor (DNeasy Blood & Tissue Kit, Qiagen, CDMX, Mexico) and from peripheral blood (Puregene Blood Kit, Qiagen, CDMX, Mex) according to manufacturer protocols. The DNA was subjected to exome purification and sequencing at the Broad´s Institute, following previously described protocols^26^. The mean coverage obtained after alignment was 89% at 20x for the germinal DNA and 88% at 20x for the tumoral DNA. Sequencing was performed using an Illumina HiSeq 2000 with the V3 Sequencing kits and the Illumina 1.3.4 pipeline (Illumina, CA). For variant calling, we used MuTect ver 1.1.4^27^ in High Confidence mode (HC) for SNV and InDelocator for small insertions and deletions. Variants were annotated with Oncotator^28^. Non-coding variants, with the exception of splice-site mutations were excluded. SNVs and InDels were further filtered by predicted protein functional impact using Combined Annotation-Dependent Depletion algorithm (CADD)^12^, with a cutoff of 1.5. Genes included in the CNV regions were filtered against a list of previously reported genes affected by CNV^17^. Germinal variants were called using Broad´s Institute Best Practices approach, where the BAM files were re-calibrated with the HaplotypeCaller using a joint genotyping approach with 60 additional normal exomes. After quality filtering we excluded variants with a Minor Allele Frequency (MAF) of 1% or greater allelic in Amerindian/European/African/Asian populations (1000 genomes, EXAC, dbSNP and local databases). We then established a tiered approach, where variants were assigned to tier 1 group if they were present in genes responsible for autosomal dominant cancer syndrome and tier 2 if have been associated with recessive cancer syndromes. All variants were manually checked with IGV.

### Transcriptome analyses

RNA was isolated using TRIzol reagent (Thermo Fisher Scientific, MA, USA). RNA with a RIN of 8 was used to construct a library using Illumina´s TruSeq RNA kit, following the manufacturer´s instructions. The paired-end library was sequenced using a GAIIx equipment (Illumina, CA) in a 72 bp configuration. After quality control and trimming, the reads were aligned with the STAR aligner^29^ and the resulting SAM was further processed with the PICARD tool^30^ to recalibrate reads. Finally, we called and filtered variants using the Haplotype Caller from GATK. Fusion transcripts were obtained with the TopHat-fusion pipeline^31^. To quantify differentially-expressed transcripts, we realigned and processed.

RNASeq data from 10 normal human fibroblasts samples^32^ (GEO dataset GSE51518 from the NCBI) together with the ovarian fibrosarcoma data and used RSEM^33^ and the R package EBSeq^34^ to normalize, quantitate and compare the expression data. microRNA analysis was performed using a miRNA 4.0 array (Affymetrix, Santa Clara, CA). A normal ovary cell line cel file was obtained from the GEO information system (GSE76449^35^) for comparison. Quality control, background subtracted, quantile normalized and log^2^- transformed using robust multi-array analysis (RMA) and differential expression analysis of the three cel files were done with Partek v 6.6. Candidate miRNAs were considered to be differentially-regulated if they presented a Fold Change >  = 2, p-values < 0.05 and FDR less than 0.05.

### Structural variants

Copy-number variants (CNV) were predicted using Control-Freec ver 8.0^36^, calculating variant changes by exon and using a breakpoint threshold of 1.5. Translocations were assessed using Delly^37^, using standard parameters.

### MSI Status

MSI status was infered from raw tumoral SNV and microInDels data using the MSIseq package^21^, using the author´s 526 tumoral exomes training database. MSI was validated using a panel of 5 markers that include BAT25, BAT26, NR21, NR22 AND NR24^38^; PCR of 5 markers in germinal and tumor sample DNA was performed as follows: denaturation 95 °C for 10 m, followed to 35 cycles (95 °C for 30 s, 50 °C for 30 s and 72° for 30 s) (Table S1). PCR products size was analyzed with the Agilent 4200 TapeStation System (Agilent Technologies).

### Sanger sequencing

DNA derived from leukocytes or tumoral tissue was subjected to PCR with the following conditions: denaturation 95 °C for 10 m, followed by 35 cycles (95 °C for 30 s, 60 °C for 30 s and 72 °C for 30 s), using the following primers with added M13 sequences to facilitate sequencing (Table S1). PCR products were gel-purified using QIAquick Gel Extraction kit (Qiagen, CA) and sequenced in a 3730XL DNA analyzer using the Big Dye direct sequencing kit (Applied Biosystems, CA).

#### Compliance with ethical standards

Ethical approval was obtained from Instituto Nacional de Medicina Genomica Research and Ethical boards, Hospital Infantil del Mexico research and ethical boards, and Federal COFEPRIS (Comision Federal para la Proteccion contra Riesgos Sanitarios). Informed consent was obtained from all participants. All procedures were carried out in accordance to the ethical guidelines of all the mentioned Research and Ethical Boards.

## Electronic supplementary material

Supplementary Information

Supplementary Data
