# Supplementary material for: Genomics of a pediatric ovarian fibrosarcoma. Association with the DICER1 syndrome
Source: Sci Rep. 2018 Feb 19;8:3252. doi: 10.1038/s41598-018-21663-9 (PMC5818504; doi:10.1038/s41598-018-21663-9)

**Genomics of a pediatric ovarian fibrosarcoma. Association with the DICER1 syndrome.**

Jorge Melendez-Zajgla, Gabriela E Mercado-Celis, Javier Gaytan-Cervantes, Amada Torres, Nayeli Belem Gabiño, Martha Zapata-Tarres, Luis Enrique Juarez-Villegas, Pablo Lezama-del-Valle, Vilma Maldonado, Karen Ruiz-Monroy, Elvia Mendoza-Caamal.

**Supplementary Tables**

Table S1. Primers used

|  | Sense | Antisense |
| --- | --- | --- |
| BAT25 | TCGCCTCCAAGAATGTAAGT | TCTGCATTTTAACTATGGCTC |
| BAT26 | TGACTACTTTTGACTTCAGCC | AACCATTCAACATTTTTAACCC |
| NR21 | TAAATGTATGTCTCCCCTGG | ATTCCTACTCCGCATTCACA |
| NR22 | GAGGCTTGTCAAGGACATAA | AATTCTGATGCCATCCAGTT |
| NR24 | CCATTGCTGAATTTTACCTC | ATTGTGCCATTGCATTCCAA |
| DICER1 Germinal insertion | GGCCAGTTCACGCTCTTCT | CCAGAAATGAAGTCTGGTCGT |

Table S2. Mutations in long non-coding RNAs

| lincRNA | Chr | Start | End | Variant | Ref. Allele | Tumor Allele |
| --- | --- | --- | --- | --- | --- | --- |
| RP11-417J8.6 | 1 | 142657355 | 142657356 | INS | - | T |
| RP11-526A4.1 | 4 | 150526021 | 150526021 | DEL | G | - |
| RP11-146I2.1 | 6 | 15021515 | 15021516 | INS | - | C |
| AC147651.1 | 7 | 534289 | 534290 | INS | - | GA |
| RP11-149P24.1 | 8 | 137130960 | 137130961 | DEL | TG | - |
| LINC00469 | 17 | 71754131 | 71754134 | DEL | TCTC | - |
| RP11-325K19.1 | 18 | 58554075 | 58554076 | DEL | GT | - |
| RP11-567M16.3 | 18 | 77408917 | 77408919 | DEL | GGT | - |

Mutated lincRNAs. Chr: Chromosome; Start and End: Gene location;

**Supplementary Figures**


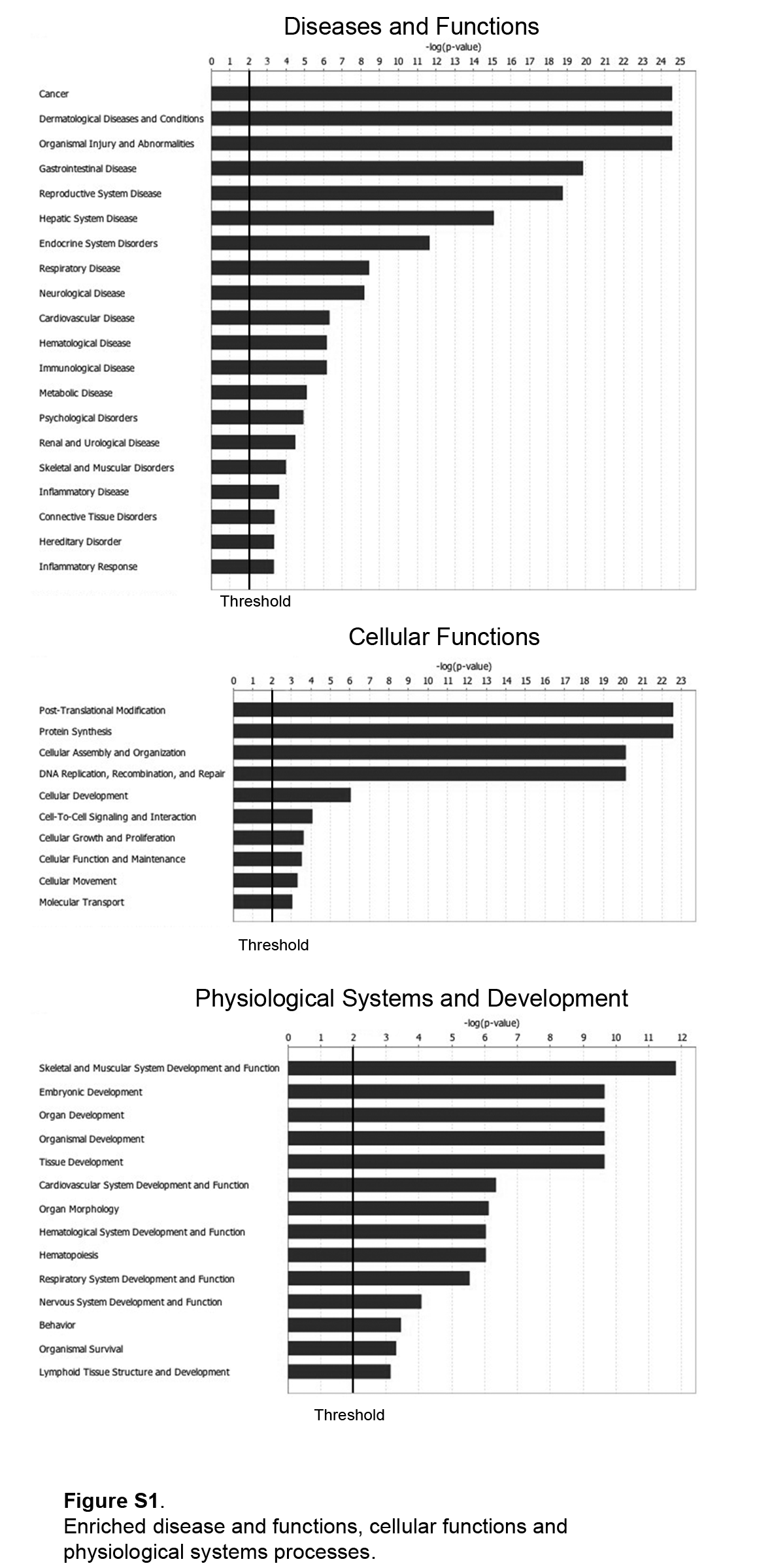


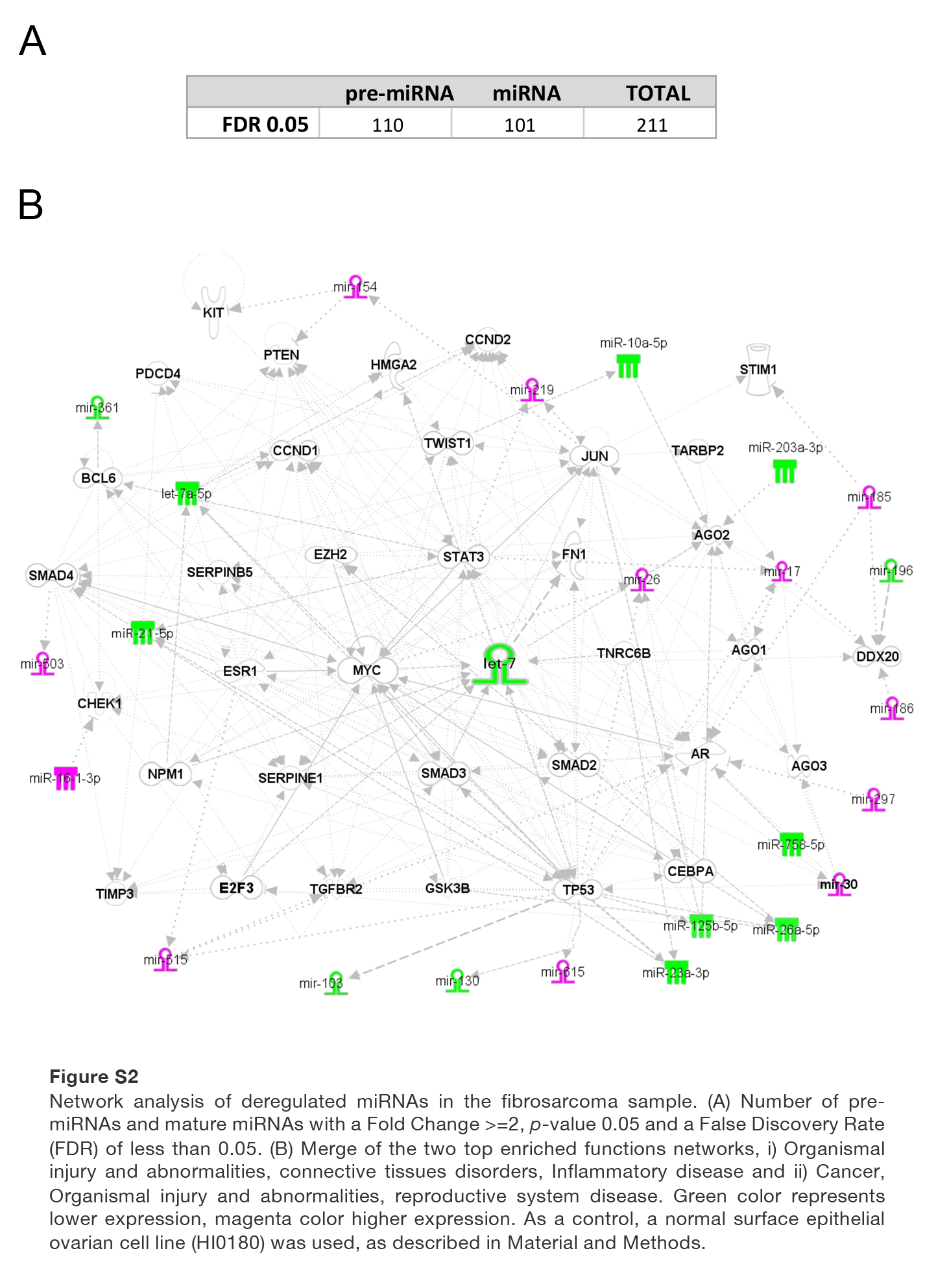

Supplement: Supplementary file 1 — Supplementary Information [file 41598_2018_21663_MOESM1_ESM.docx]
